# Simultaneous Measurement of Temperature and Refractive Index Using Michelson Interferometer Based on Waist-Enlarged Fiber Bitaper

**DOI:** 10.3390/mi13050658

**Published:** 2022-04-20

**Authors:** Na Zhao, Zelin Wang, Zhongkai Zhang, Qijing Lin, Kun Yao, Liangquan Zhu, Bian Tian, Libo Zhao, Ping Yang, Zhuangde Jiang

**Affiliations:** 1 State Key Laboratory for Manufacturing Systems Engineering, Xi’an Jiaotong University, Xi’an 710049, China; zn2020@xjtu.edu.cn (N.Z.); wzl15086927209@stu.xjtu.edu.cn (Z.W.); yao_kun@outlook.com (K.Y.); zhuliangquan@stu.xjtu.edu.cn (L.Z.); t.b12@mail.xjtu.edu.cn (B.T.); libozhao@xjtu.edu.cn (L.Z.); ipe@xjtu.edu.cn (P.Y.); zdjiang@xjtu.edu.cn (Z.J.); 2 Collaborative Innovation Center of High-End Manufacturing Equipment, Xi’an Jiaotong University, Xi’an 710054, China

**Keywords:** fiber-optic sensors, temperature, refractive index, multi-parameter sensing, Michelson interferometer

## Abstract

An all-fiber temperature and refractive dual-parameter-sensing Michelson interferometer is designed based on the waist-enlarged bitaper. At 5 mm from the fiber end, the waist-enlarged bitaper is manually spliced and the probe is formed. Since the input light encounters the waist-enlarged bitaper, it will excite high-order modes to transmit in the fiber cladding, and there will be an optical path difference between the basic mode and the higher-order mode. The light transmitted in the core and cladding is reflected upon encountering the fiber end face and the interference occurs due to the optical path difference between basic mode and higher-order mode. Changes in temperature and refractive index at the fiber probe can be detected by monitoring the interference fringes. The refractive response sensitivity is −191.06 dBm/RIU from 1.351 RIU to 1.4027 RIU, and the temperature response sensitivity is 0.12 nm/°C from 11 °C to 98 °C. Through the sensitivity matrix equation, the superimposed refractive index and temperature signals can be effectively demodulated. The sensor has the advantages of multi-parameter measurement, compact structure, low cost, easy fabrication and high reliability.

## 1. Introduction

Temperature and refractive index are important parameters in the biochemical, food processing, pharmaceutical, oil extraction and biochemical measurement fields. Traditional electrical sensors find it difficult to meet the sensing requirements of these fields under severe environmental factors such as corrosion resistance, oxidation resistance and electromagnetic interference resistance. The optical fiber sensor performs sensing measurement based on optical signals, which can overcome the sensing problems such as oxidation resistance and corrosion resistance that electrical sensors cannot deal with, and has the advantages of compact structure, multi-parameter sensing measurement and high sensitivity, which make the optical fiber sensor a multi-parameter sensor.

In recent years, various fiber-optic sensors have been reported, such as long-period fiber gratings [1,2], fiber Bragg gratings [3,4,5,6], Mach–Zehnder interferometer [7,8,9,10,11] and Michelson interferometer [12,13,14,15,16,17,18,19], etc. In 2020, JENS HØVIK et al. [1] of Norwegian University developed a wavelength refractive index sensor based on long-period grating, and a response sensitivity of 5078 nm/RIU was measured from 1.33 RIU to 1.34 RIU. Subsequently, Yang H et al. [3] of Nanchang University developed a refractive index sensor using fiber Bragg grating from the perspective of intensity modulation, and a maximum refractive index sensitivity of −134.174 dB/RIU was obtained. In 2021, Wang, S. et al. [12] of Nanyang Technological University in Singapore developed a fiber tip Michelson interferometer, which partially filled with polymer glue in the suspended core fiber, and achieved high-sensitivity temperature sensing of −164 pm/°C from 25 °C to 60 °C. In the same year, Zhao, Y. et al. [13] from Tsinghua University proposed a polarization-maintaining fiber Sagnac temperature sensor based on combination with a fiber-optic Michelson structure, and the temperature sensitivity is 78.984 nm/°C from 31 °C to 35 °C; however, the reduction of the overall structure of the sensor still needs continuous exploration. The above studies have not covered the measurement of temperature and refractive index dual-parameter sensing, so multi-parameter sensing needs further research.

In 2019, Gao, X. et al. [7] of Beijing Jiaotong University measured the refractive index and temperature at the same time based on MZI combined with coreless fiber and few-mode fiber; the refractive index sensitivity from 1.3707 RIU to 1.39809 RIU was 97 pm/RIU, 22.9 pm/RIU and 24.6 pm/RIU, respectively, and the temperature sensitivity from 35 °C to 55 °C was 162 pm/°C, 162 pm/°C and 194 pm/°C, respectively. In order to further improve the temperature and refractive index sensing performance of the sensor, in 2021, Siti Mahfuza, Saimon et al. [6] from Universiti Malaysia proposed a novel fiber-optic sensor, which is composed of a section of single-mode silica rod–single-mode fiber structure cascaded to fiber Bragg grating, by monitoring the transmission spectrum; a sensitivity of 108.07 dBm/RIU in the refractive index range of 1.45 RIU to 1.531 RIU and a sensitivity of 9.31 pm/°C from 35 °C to 85 °C was measured. In the same year, Wu, B. et al. [8] proposed a curved-core-shifted coaxial Mach–Zehnder interferometer with a bending radius of 35.64 mm; the highest refractive index sensitivity in the range of 1.333 RIU to 1.373 RIU was −44.55 nm/RIU, and the highest temperature sensitivity in the temperature range of 25 °C to 60 °C was 0.0799 nm/°C. The above structures can realize temperature and refractive index sensing, but they are transmissive structures that are not easy to transform into probe structures. Therefore, the Michelson interferometers which can be made into probe structures have become the focus of research.

In 2019, Wang, J. et al. [14] fabricated a Michelson interferometer by splicing a single-mode fiber and a hollow silica tube, with a refractive index and temperature sensitivity of 8.1498 rad/RIU from 1.331 RIU to 1.387 RIU, and −0.05 rad/°C from 20 °C to 90 °C. In 2020, Qi K et al. [15] of Harbin Institute of Technology proposed a fiber-optic Michelson interferometer based on a three-microsphere array, which measured a maximum temperature sensitivity of 115.3 pm/°C from 20 °C to 90 °C and a refractive sensitivity of −56.63 nm/RIU in the range of 1.3335 RIU to 1.406 RIU; the fabrication repeatability of the three microsphere structures of the sensor is difficult to control, and the repeatability of the process needs to be further enhanced. In 2021, Zheng J et al. [16] proposed a thin-waist-cone Michelson interferometer, and measured the temperature sensitivity of wavelength demodulation as being 8.4 pm/°C, and the refractive index sensitivity of intensity demodulation as being −145.54 dB/RIU. In the same year, Zhang Y et al. [17] designed a new L-type Michelson interferometer based on the method of flame firing. The refractive index response sensitivity was −131.0 nm/RIU from 1.3430 RIU to 1.3927 RIU, and the temperature response sensitivity was 94.17 pm/°C from 30 °C to 100 °C. The sensor is greatly affected by the position of the flame intensity during the production process, and needs to be further improved in terms of repeatability. The waist-enlarged fiber bitaper can be used as a coupling point [20,21]. Yanhong Liang et al. [22] from Zhejiang University developed a novel optical fiber sensor, based on the waist-enlarged fiber bitaper and the polyvinyl alcohol film, where the refractive index variance ranges from 1.49 to 1.34, the ambient humidity increases from 20%RH to 95%RH, and a sensitivity up to 1.2 dB/%RH can be achieved.

In this paper, a Michelson interferometer based on the waist-enlarged fiber bitaper is developed, which realizes the simultaneous sensing of temperature and the refractive index. The sensor has a compact structure, and a thick waist-cone structure is directly fabricated on the optical fiber by manual welding, which eliminates the process of fiber cutting and alignment welding, and reduces the manufacturing steps of the sensing probe. The spectrum of the optical signal is analyzed, and the mode distribution diagram of the optical fiber section is given. The intensity and wavelength changes of the spectra are analyzed, and the temperature and refractive index are measured using the sensitivity matrix equation.

## 2. Principle and Design 

**Figure 1:**
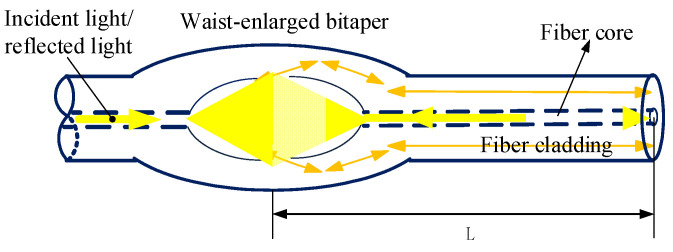
Schematic diagram of Michelson interferometer.

The schematic diagram of the fiber Michelson interferometer is shown in Figure 1, where the distance *L* between the waist-enlarged bitaper and the right end face is the length of the sensing arm. When the input light is transmitted from the left to the position of the waist-enlarged bitaper, the higher-order modes are excitation in the cladding. The lower-order fundamental modes continue to propagate in the core. When the light transmitted in the fiber core and fiber cladding encounters the reflective end face, it is reflected and returns along the original path. When the waist-enlarged bitaper is encountered for the second time, the different orders of light are coupled together and form interference fringes.

The formation of interference fringes is caused by the optical path difference of the transmitted light in the fiber core and cladding. We can first list the optical path difference equation, as follows [23,24]:

(1) $$\Delta \phi =\frac{4\pi }{\lambda }\left(n_{m}-n_{core}\right)L\;\;\;\;m=1,2,3\ldots \ldots$$

where *n_m_* is the effective refractive index of the optical fiber cladding, *n_core_* is the effective refractive index of the fiber core, *L* is the length of Michelson interferometer and λ is the wavelength. 

If we define the intensities of fiber core mode and fiber cladding mode as $I_{core}$ and $I_{m}\left(RI\right)$, the effective refractive index difference is $\Delta n_{eff}$ and the initial phase is $\phi_{0}$; the intensity of the transmission spectrum can be defined as [25,26]:

(2) $$I\left(\lambda \right)=I_{core}+\sum_{m}I_{m}\left(RI\right)+\sum_{m}2{\left[I_{core}\times I_{m}\left(RI\right)\right]}^{\frac{1}{2}}\cdot \cos\left(\frac{4\pi \Delta n_{eff}L}{\lambda }+\phi_{0}\right)$$

When the external refractive index changes, the intensity of the reflected light will change. Changes in light intensity are related to changes in the external refractive index. Assuming that the initial phase is equal to 0, the change in the interference intensity can be described as [27]:

(3) dIdRI=∑mdIm(RI)dRI+Icore⋅∑m[IcoreIm(RI)]12⋅dIm(RI)dRI⋅cos{4πλ[Icore−Im(RI)]⋅L}+∑m[IcoreIm(RI)]12⋅sin{4πλ[Icore−Im(RI)]⋅L}⋅8πλ⋅L⋅dnm(RI)dRI

The generation of the interference spectrum is mainly caused by the phase difference, the phase condition represented by the cosine phase of Equation (2); therefore, the spectral phase at a certain moment can be expressed as the following equation:

(4) $$\phi =\frac{4\pi n_{core}L}{\lambda }+\phi_{0}$$

When the temperature changes, the length and refractive index of the fiber material will change, and the center wavelength will also drift accordingly. Assuming that when the temperature increases by Δ*T*, the cavity refractive index of the fiber sensor changes from *n* to $n_{core}+n_{core}\xi \Delta T$, the length changes from L to L+LαΔT, and the center wavelength λ will drift to λ+Δλ, so [23,25]:

(5) $$\phi =\frac{4\pi (n_{core}+n_{core}\xi \Delta T)(L+L\alpha \Delta T)}{\lambda +\Delta \lambda }$$

In the equation: Δ*T*—temperature change; *ξ*—thermo-optical coefficient. The change of wavelength with temperature has little to do with the initial phase. In order to simplify the equation, we set the initial phase *ϕ*_0_ as 0; then, from Equations (4) and (5), we can obtain:

(6) $$\Delta \lambda =\lambda \left(\xi \Delta T+\alpha \Delta T+\xi \alpha \Delta T^{2}\right)$$

Because the wavelength and temperature change is very small, the equation can be approximated to Equation (7):

(7) $$\frac{\Delta \lambda }{\Delta T}\approx \frac{d\lambda }{dT}=\lambda \left(\xi +\alpha \right)$$

Equation (7) characterizes the change in wavelength with temperature. The thermal expansion coefficient and the thermo-optic coefficient are constants. Therefore, it can be drawn theoretically that when the monitoring wavelength is constant, the temperature response sensitivity is a constant; in other words, the change of wavelength is approximately linear with the change of temperature, which can be confirmed by the following temperature experiments.

**Figure 2:**
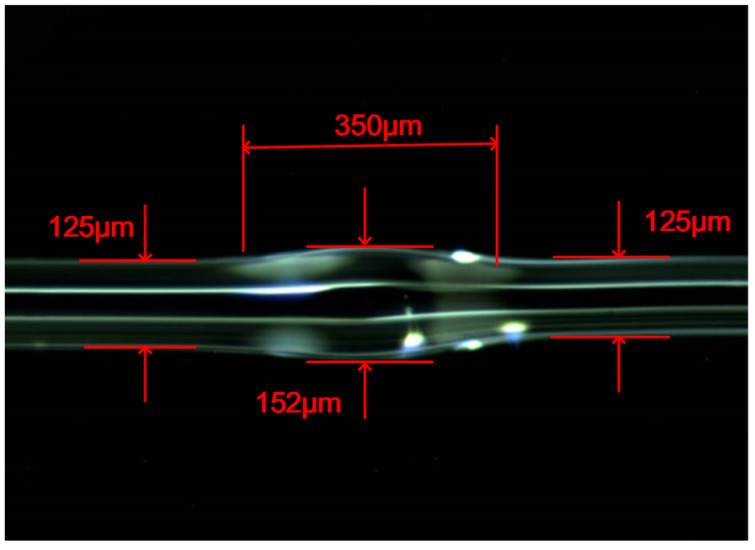
The optical fiber waist-enlarged bitaper under microscope.

A fusion splicer (Furukawa, S177B, Tokyo, Japan) is used to fabricate the fiber Michelson, and the fiber used for the sensor is a common single-mode fiber with core and cladding diameters of 9 µm and 125 µm, respectively. A fiber cleaver (Furukawa, S325, Tokyo, Japan) is used to cut the fiber end face flat to obtain a reflective end face with high reflectivity. The part between the waist-enlarged bitaper and the reflective end face is the sensing arm. According to the required length of the sensing arm, the position of the waist-enlarged bitaper is selected and the manual welding program is set to make the waist-enlarged fiber bitaper. Through a large number of experimental comparisons, it is found that if the waist size of the waist-enlarged bitaper is too small, the contrast of the generated interference fringes will become smaller; and if the bitaper is too large, the overall intensity of the spectrum will be reduced. Therefore, it is necessary to restrict the size of the waist-enlarged bitaper structure within a certain range, and an intermediate size is selected in the manufacturing process. The parameters in the specific production process are as follows. The discharge cleaning is set to 50 ms, the discharge duration is set to 1100 ms, the discharge intensity is set to 155 xmW and the push distance is set to 140 μm. Due to the high discharge intensity and the large advancing distance, the diameter of the set point will slowly expand. Figure 2 is a 90-times magnified photo of the optical fiber waist-enlarged bitaper under the microscope (Olympus, SZ61, Center Valley, PA, USA). It can be seen from the figure that the diameter of the welding point is enlarged from 125 μm to about 152 μm, and the length of the optical fiber waist-enlarged bitaper is about 350 μm.

**Figure 3:**
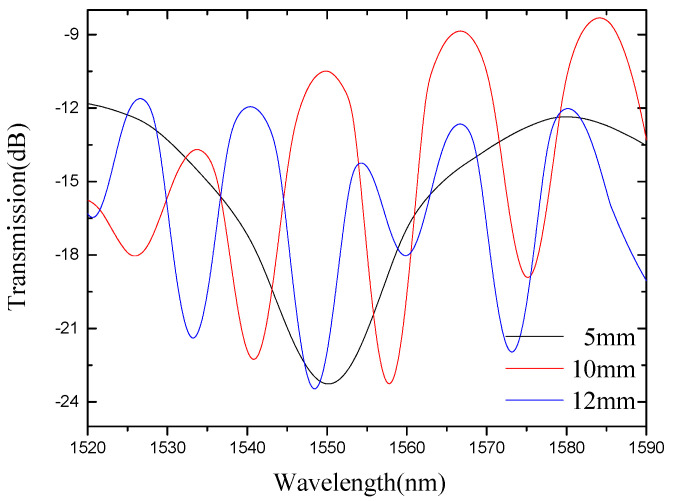
Interferometric spectra of sensors with different lengths.

**Figure 4:**
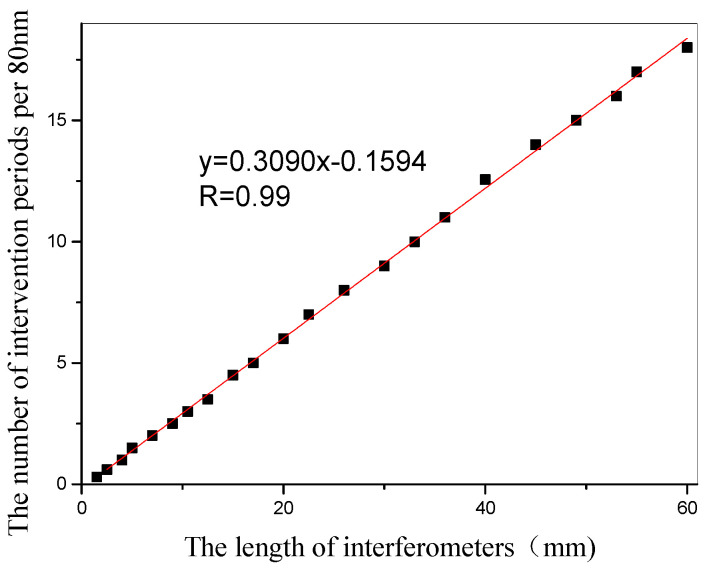
Relationship between interference period and interference arm length in the 80 nm spectral range.

Figure 3 shows the interference fringes corresponding to sensors with different lengths. It can be seen from the figure that the contrast of the interference fringes is high, and the interference peaks and interference valleys are clearly visible. By fabricating a large number of sensors, we summarize the relationship between the interference arm length and the interference period per 80 nm spectral range. It can be seen from Figure 4 that as the length of the interference arm increases, the spectrum in the unit spectral range increases. In other words, as the interference arms grow, the interference fringes become denser.

**Figure 5:**
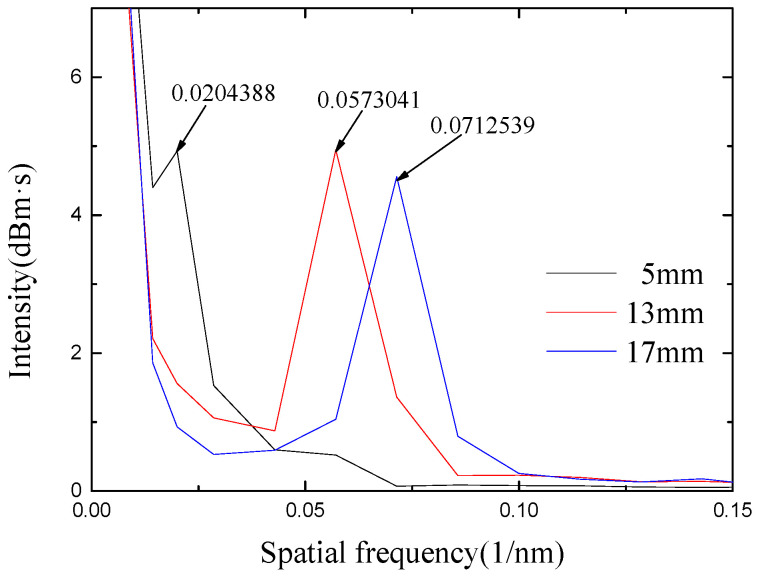
Spatial spectrum analysis of sensors with different lengths.

The spectrum is converted to a spatial spectrum by a fast Fourier transform (FFT), as shown in Figure 5. It can be seen that the high-order spectra corresponding to sensors of different lengths have only one main peak. For sensors with interference lengths of 5 mm, 13 mm and 17 mm, the positions of the main peaks are at 0.0204388 nm^−1^, 0.0573041 nm^−1^ and 0.0712539 nm^−1^, respectively. Based on Taylor expansion, the phase ϕ can be defined as [24]:

(8) $$\phi \approx \phi_{0}-\frac{2\pi \Delta \lambda }{\lambda^{2}}\Delta n_{eff}\cdot 2L$$

Meanwhile, the phase part of the interference wavelength can be expressed as a cosine equation, as shown below [28].

(9) cosϕ=cos(2πξΔλ)

Assuming the initial phase $\phi_{0}$ is equal to 0, we can obtain the spatial frequency *ξ* based on Equations (8) and (9), as shown in Equation (10).

(10) $$\xi =\frac{2}{\lambda^{2}}\Delta n_{eff}\cdot L$$

By transforming Equation (10), it can be obtained that the refractive index difference is as follows.

(11) $$\Delta n_{eff}=\frac{\xi \cdot \lambda^{2}}{2L}$$

**Figure 6:**
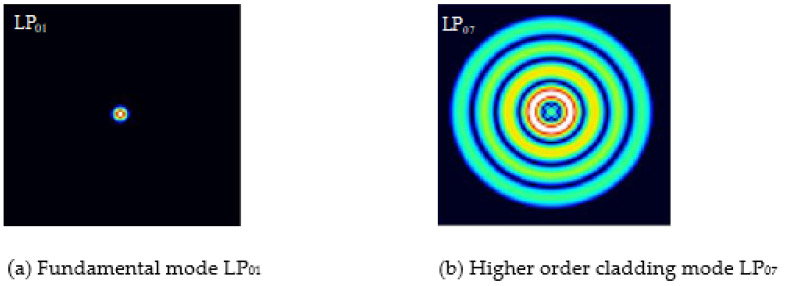
Optical mode distribution diagram of optical fiber section.

The C + *L* band is selected in the experiment; the center wavelength is around 1550 nm, and the lengths of the interference arms are 5 mm, 13 mm and 17 mm, respectively. Substituting each parameter into Equation (11), the refractive index difference Δ*n_eff_* between modes can be calculated to be 0.004910422 dB·s, 0.005295119 dB·s and 0.005034926 dB·s, respectively. The relationship between Δ*n_eff_* and different modes is analyzed by OptiFiber software, as shown in Table 1. By analyzing the effective refractive index difference between the higher-order mode and the fundamental mode, the experimentally obtained refractive index difference is closest to the difference between LP_01_ and LP_07_. Among them, LP_01_ is mainly transmitted in the core, and LP_07_ is mainly transmitted in the cladding. The optical mode distribution of the fiber-truncated surface of the fundamental mode LP_01_ and high-order LP_07_ mode given by software simulation is shown in Figure 6.

## 3. Experiment and Discussion

### 3.1. Refractive Index Response Experiment

**Figure 7:**
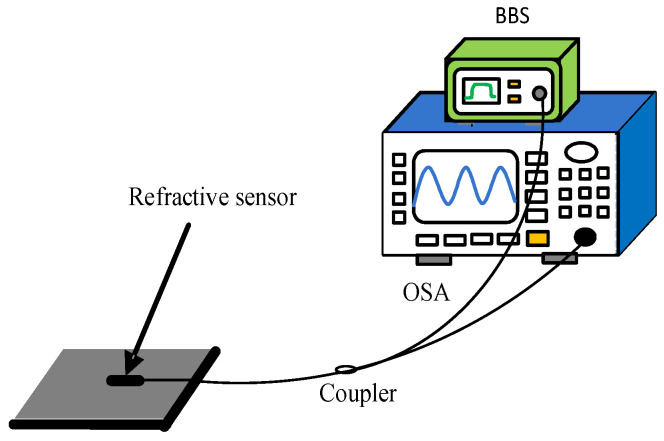
Refractive index sensing experimental device schematic diagram.

As shown in Figure 7, the fiber Michelson interferometer measuring system is composed of an optical spectrum analyzer (OSA, Anritsu, MS9740A, Kanagawa, Japan) and a broadband light source (BBS, Lightcomm, ASE-CL, Shenzhen, China). The resolution of the OSA is set to be 0.02 nm, and the bandwidth of BBS is 80 nm. Sugar solutions with different refractive indices are prepared as samples for measurement, which are set as 1.351 RIU, 1.357 RIU, 1.369 RIU, 1.369 RIU, 1.378 RIU, 1.3872 RIU, 1.3941 RIU and 1.4027 RIU. Further, the refractive index solutions are verified by Abbe refractometer. During the specific experiment, a single concentration of sugar solution is dropped on the sensor head, and the OSA is utilized to record the transmission spectral response. Before the next measurement of the sugar solution, the sensor head is rinsed repeatedly with water and allowed to dry. In addition, the ambient temperature is kept at 11 °C to minimize the cross effects of temperature.

**Figure 8:**
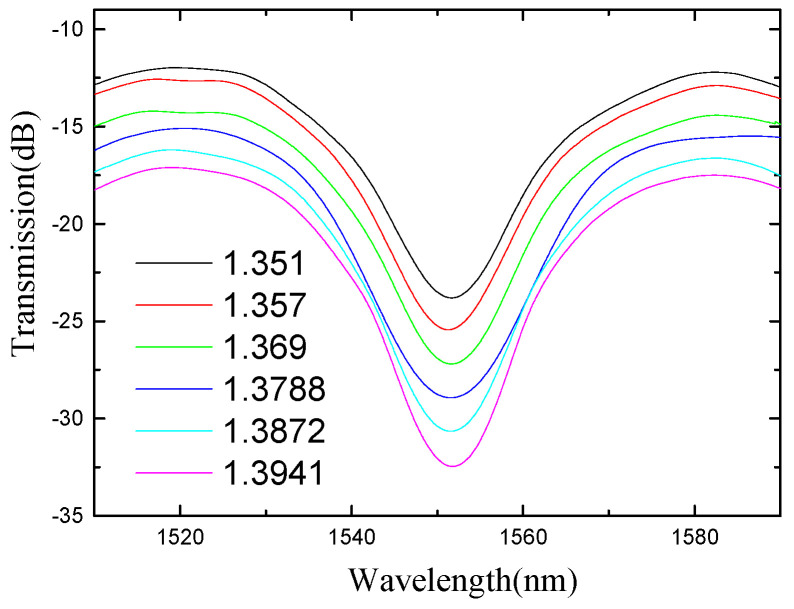
The spectra under different ambient refractive indices.

**Figure 9:**
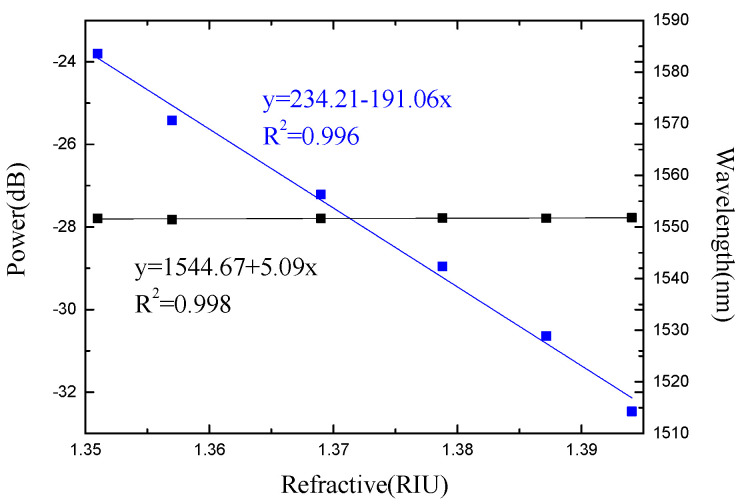
The refractive index response of the Michelson interferometer.

As the refractive index of the liquid increases, the intensity and wavelength of the spectral valleys change accordingly, as shown in Figure 8. The blue dots are the measured intensity data, and the black dots are the measured wavelength data. The fitted line is obtained by the least squares fitting method. A linear decrease in the power of the trough can be observed. With the refractive index changes from 1.351 RIU to 1.4027 RIU, an intensity response sensitivity of −191.06 dB/RIU and a wavelength response sensitivity of 5.09 nm/RIU are obtained, as shown in Figure 9.

In addition, the change of air humidity can also be attributed to the small change of the air refractive index, so we also experimentally designed a humidity experiment. In the humidity range of 20% to 80%, the interference spectrum of the sensor hardly changes with humidity. This can be attributed to the fact that the refractive index change is too small; the total change of air refractive index with air humidity is less than 0.00005 RIU [29]. The spectral change is too low, and the demodulation equipment can no longer distinguish it.

### 3.2. Temperature Response Experiment

**Figure 10:**
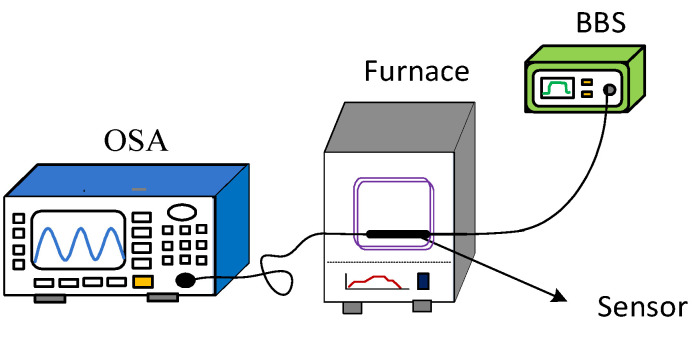
Temperature sensing experimental device schematic diagram.

**Figure 11:**
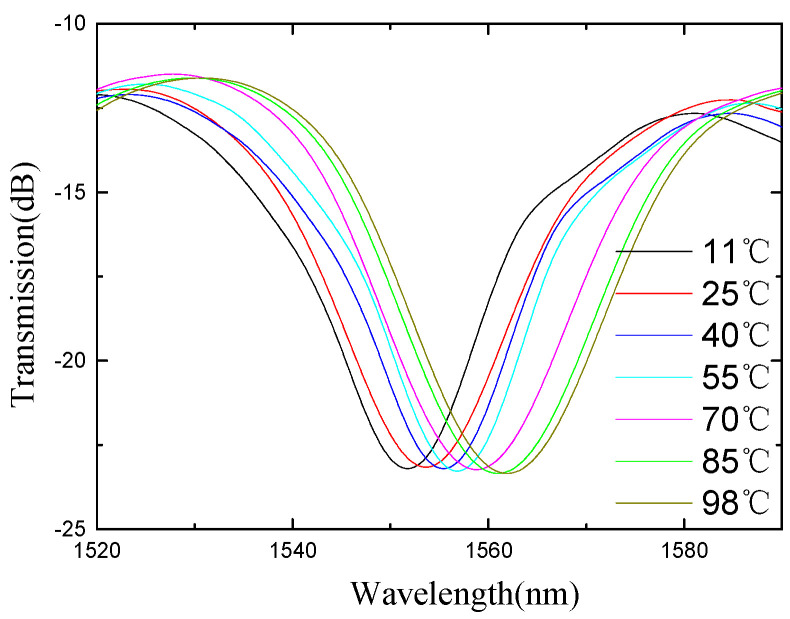
The spectra under different ambient temperatures.

The schematic diagram of the temperature experimental device is shown in Figure 10. The light emitted by the ASE light source is transmitted into the sensor, and the MS740A spectrometer (Anritsu, MS9740A, Kanagawa, Japan) is used to measure the reflection spectrum of the sensing system. The temperature control system is a high-temperature muffle furnace (Omega, Biel/Bienne, Switzerland), and the temperature control accuracy is 1 °C. As the temperature increases, the spectrum of the fiber Michelson shifts due to the thermo-optic and thermal expansion effects, as shown in Figure 11. It can be seen from the figure that the sensor is sensitive to temperature changes and can be designed as a temperature sensor.

**Figure 12:**
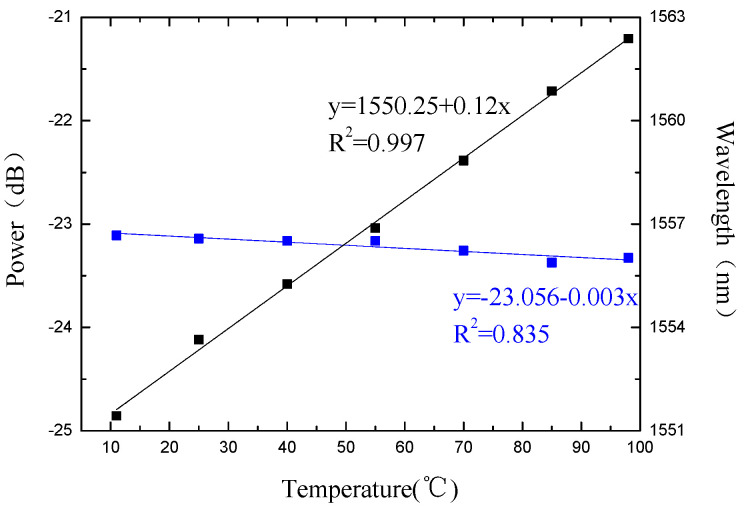
The temperature response of the Michelson interferometer.

The trough around 1555 nm is monitored and used to analyze the temperature response sensitivity of the fiber Michelson interferometer, as shown in Figure 11. The shift of the trough intensity and wavelength with temperature is analyzed as shown in Figure 12. The blue dots and blue lines are the measured intensity data and the fitted line, and the black dots are the measured wavelength data and the fitted line. Temperature sensitivities are −0.011 nm/°C and –0.009 dB/°C from 30 °C to 90 °C.

(12) $$\left[\begin{array}{c} \Delta P\\ \Delta \lambda \end{array}\right]=\left[\begin{array}{cc} K_{nP} & K_{TP}\\ K_{n\lambda } & K_{T\lambda } \end{array}\right]\left[\begin{array}{c} \Delta n\\ \Delta T \end{array}\right]$$

In Equation (12), Δ*n* and Δ*T* are the refractive index and temperature changes, respectively. *K_nP_* and *K_nλ_* are the intensity and wavelength response sensitivities when the refractive index changes. *K_TP_* and *K_Tλ_* are the intensity and wavelength response sensitivities when temperature changes. Multiplying both sides of the Equation by the inverse of the sensitivity matrix, Equation (13) can be obtained [30].

(13) $$\left[\begin{array}{c} \Delta n\\ \Delta T \end{array}\right]={\left[\begin{array}{cc} K_{nP} & K_{TP}\\ K_{n\lambda } & K_{T\lambda } \end{array}\right]}^{-1}\left[\begin{array}{c} \Delta P\\ \Delta \lambda \end{array}\right]$$

The corresponding relationship between the experimentally measured sensitivity coefficient of each parameter and each parameter of the matrix equation is as follows: *K_nP_* = −191.06 dB/RIU, *K_nλ_* = −5.09 nm/°C, *K_TP_* = −0.003 dB/RIU, *K_Tλ_* = 0.12 nm/°C. Substituting the measurement data into Equation (13), Equation (14) can be obtained. Through the analysis of the spectrum, the changes of the refractive index and temperature value of the detection environment can be obtained based on Equation (14).

(14) $$\left[\begin{array}{c} \Delta n\\ \Delta T \end{array}\right]={\left[\begin{array}{cc} -191.06 & 5.09\\ -0.003 & 0.12 \end{array}\right]}^{-1}\left[\begin{array}{c} \Delta P\\ \Delta \lambda \end{array}\right]$$

## 4. Conclusions

In this paper, a waist-enlarged bitaper Michelson interferometer for multi-parameter sensing is proposed. By comparing the spectra of probes with different lengths, it is found that as the length of the sensor probe increases, the distance between the two valleys of the interference spectrum becomes smaller. The experimental analysis of the temperature-refractive index characteristics of the sensor shows that the sensor has a wavelength response sensitivity of 0.12 nm/°C and an intensity response sensitivity of −0.003 dBm/°C in the range of 11 °C to 98 °C. In the refractive index range of 1.351 RIU to 1.4027 RIU, the wavelength response sensitivity is 5.09 nm/RIU, and the intensity response sensitivity is −191.06 dBm/RIU. The measurement results prove that this type of optical fiber sensor can measure various external parameters according to the change of spectral intensity and wavelength, combined with the sensitivity matrix equation. In the process of food processing and pharmaceutical production, the temperature and refractive index of the product can be obtained through sensor monitoring in a timely manner, and then the product quality can be controlled. At the same time, it can also monitor the temperature and refractive index in real time during oil exploration to obtain the temperature of underground oil and the quality of crude oil. In short, the sensor has the advantages of simple structure, high sensitivity and multi-parameter measurement, and has certain application value.

## Figures and Tables

**Table 1:** Relationship between mode order and refractive index difference Δ*n_eff_*.

X	*n*_LP0*x*_	Δ*n_eff_*
1	1.4658679	0
2	1.4627498	0.0031181
3	1.4625833	0.0032846
4	1.4623038	0.0035641
5	1.4619147	0.0039532
6	1.4614194	0.0044485
7	1.4608213	0.0050466
8	1.4601245	0.0057434
9	1.4593324	0.0065355
